# Autophagy flux induced by ginsenoside-Rg3 attenuates human prion protein-mediated neurotoxicity and mitochondrial dysfunction

**DOI:** 10.18632/oncotarget.13730

**Published:** 2016-11-30

**Authors:** Ji-Hong Moon, Ju-Hee Lee, You-Jin Lee, Sang-Youel Park

**Affiliations:** ^1^ Biosafety Research Institute, College of Veterinary Medicine, Chonbuk National University, Iksan, Jeonbuk, South Korea

**Keywords:** ginsenoside Rg3, autophagy, neuron, mitochondria, prion, Gerotarget

## Abstract

Mitochondrial quality control is a process by which mitochondria undergo successive rounds of fusion and fission with dynamic exchange of components to segregate functional and damaged elements. Removal of mitochondrion that contains damaged components is accomplished via autophagy. In this study, we investigated whether ginsenoside Rg3, an active ingredient of the herbal medicine ginseng that is used as a tonic and restorative agent, could attenuate prion peptide, PrP (106-126)-induced neurotoxicity and mitochondrial damage. To this end, western blot and GFP-LC3B puncta assay were performed to monitor autophagy flux in neuronal cells; LC3B-II protein level was found to increase after Rg3 treatment. In addition, electron microscopy analysis showed that Rg3 enhanced autophagic vacuoles in neuronal cells. By using autophagy inhibitors wortmannin and 3-methyladenine (3MA) or autophagy protein 5 (Atg5) small interfering RNA (siRNA), we demonstrated that Rg3 could protect neurons against PrP (106-126)-induced cytotoxicity via autophagy flux. We found that Rg3 could also attenuate PrP (106-126)-induced mitochondrial damage via autophagy flux. Taken together, our results suggest that Rg3 is a possible therapeutic agent in neurodegenerative disorders, including prion diseases.

## INTRODUCTION

Prion diseases or transmissible spongiform encephalopathies (TSEs) are inevitably lethal neurodegenerative conditions that can influence humans and various kinds of animals [1]. They may present with certain morphological and pathophysiological features that parallel those of other progressive encephalopathies, including Alzheimer's disease and Parkinson's disease [2]. PrPSc, a scrapie isoform of the prion protein, was originally defined as an aggregated protease-resistant prion protein. However, it was later shown that PrPC, a cellular prion protein, may undergo disease-associated and diagnostically important modifications without becoming protease-resistant (protease-sensitive PrPSc, or sPrPSc) [3]. If the prion consists entirely of PrPSc, it may propagate by template-directed refolding or nucleation [4].

PrP (106-126), a specific peptide of PrPSc, was implemented for experimental application of PrPSc in this research [5]. PrP (106–126) includes the ability to induce apoptosis in neurons and reproduces β-sheet-rich structure, amyloidogenesis, and neurotoxic effects [5–8]. The biological activity of the 106-126 sequence has been further proved by the observation that this fragment has AGAAAAGA sequence (amino acids 113 to 120) shown to have specific region within PrP molecules in many kinds of species [9].

Autophagic dysfunction, which leads to the formation of giant autophagic vacuoles in experimental scrapie in hamsters, is associated with prion diseases [10]. The term “autophagy”, meaning “eat self”, was first coined by Christian de Duve generally based on the observation of mitochondrial and cellular component degradation within lysosomes [11]. Upon induction, a portion of cytoplasm is enclosed, resulting in the enlarged autophagosome that subsequently combines with lysosome to form autolysosomes [13]. Autophagosome formation requires localization and aggregation of LC3, microtubule-associated protein light chain 3, on its membrane. LC3, which undergoes lipidation, is therefore considered as a crucial marker of autophagy. LC3 is recruited to make the phagophore called autophagosomes where it is essential for membrane elongation and closure [14]. SQSTM1/p62 (Sequestosome-1) protein is a link between LC3 and ubiquitinated substrates and is initially incorporated into autolysosomes but is eventually degraded by them [15]. LC3B is an alternative splicing variant of LC3 and a member of the LC3 family, which includes LC3A, LC3B, and LC3C. Analysis of subcellular localization showed that both LC3A and LC3B are colocalized with LC3 and are associated with autophagic membranes [16].

The neuroprotective effects induced by activation of autophagy are associated to mitochondrial turnover [17, 18]. Autophagy can reduce the quantity of abnormal prion proteins [19]. The participation of the lysosomal system in programmed cell death has been well accepted recently, although its exact role remains controversial [20]. In cancer cells, autophagy has been shown to induce apoptosis in several cases [21, 22]. Autophagy has ability to sustain the viability of cells with defective apoptotic mechanisms [22–24].

Ginsenoside-induced regulation of ion channels could protect against excitatory neurotransmitters both *in vitro* and *in vivo* [25–28]. Panax ginseng includes more than 30 types of active components and it is useful as a tonic in customary medicine [29]. Rg3, one of the main active ingredients in P. ginseng, is well-known not only as a tonic but also as a restorative agent [30]. Some studies have shown that Rg3 has impressive neuroprotective effect against focal cerebral ischemic damage [31, 32]. It has been reported that ginsenoside Rg3 can also preserve mitochondrial function and energy status in rat brain during ischemia/reperfusion [33]. Additionally, it is well established that mitochondria play central roles in apoptosis caused by many chemotherapeutic agents [34].

However, the relationship between Rg3 and the mitochondria pathway in prion disease remains unclear. Therefore, we hypothesized that Rg3 could protect neurons against prion-induced mitochondrial dysfunction and neurotoxicity via autophagy activation. We used autophagy inhibitors, 3-methyladenine (3-MA) and wortmannin, and small interfering RNA (siRNA) tools to study the effect of autophagy flux. 3-MA and wortmannin have been widely used as phosphoinositide 3-kinase (PI3K) inhibitors [35, 36]. They have been proposed to suppress autophagy by inhibiting the class III PI3K to block the production of phosphatidylinositol 3-phosphate (PI3P), which is essential for the initiation of autophagy [37, 38]. With the aim of identifying the mechanism underlying the neuroprotective effect of Rg3, in this study, we investigated whether Rg3 induced autophagy flux in neuronal cells.

We observed that Rg3 dose-dependently induced autophagy flux in neuronal cells. The data generated in this study support our hypothesis that autophagy flux induced by Rg3 may have therapeutic implications for prion diseases.

## RESULTS

### Effect of Rg3 on prion protein-induced cellular neurotoxicity

**Figure 1:**
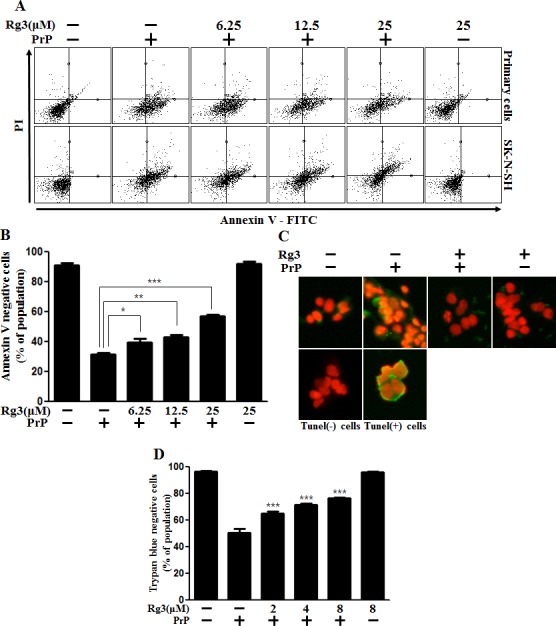
Rg3 inhibits PrP (106-126)-induced cytotoxicity in neuronal cells (**A**) Primary neuronal cells were pretreated with Rg3 (6 h) at different concentrations and then exposed to 100 μM PrP (106-126) for 12 h. Cell viability was determined with annexin V assay. Cells were treated with FITC-annexin V that could bind to phosphatidylserine on the plasma membrane during apoptosis. (**B**) Bar graph indicating the averages of annexin V-negative cells of primary neuron cells. (**C**) Representative immunofluorescence images of TUNEL-positive (green) cells of primary neuron cells at 12 h after exposure to 100 μM of PrP (106–126) in the presence or absence of Rg3 (6 h). Cells were counterstained with PI (red) to show nuclei staining. (**D**) Cell viability of primary neuron cells was measured by trypan blue dye exclusion assay. *p < 0.05, **p < 0.01, *** *p* < 0.001: Significant differences between the control group and treatment group. PrP: PrP (106-126).

We investigated the influence of Rg3 on PrP (106-126)-induced neurotoxicity in primary neuronal cells and a neuroblastoma cell line by using annexin V assay. Primary neurons were treated with either PrP (106-126) alone or in combination with Rg3. In primary neurons, the viability of only PrP (106-126)-treated cells were reduced by about 35% compared to that of controls. However, the viability of PrP (106-126)-treated cells increased upon exposure to Rg3. Rg3 attenuated PrP (106–126)-induced neuronal apoptosis (Figure 1A and 1B). In primary neurons, Rg3 reduced DNA strand breakage caused by PrP (106-126) (Figure 1C). Further analysis with trypan blue to test dose dependency of the response to Rg3 indicated that in primary neurons, Rg3 inhibited PrP (106-126)-induced apoptosis in a dose-dependent manner (Figure 1D). Thus, these results suggest that Rg3 protects mouse primary neurons against PrP (106-126)-induced cytotoxicity.

### Rg3 upregulates autophagy flux in neuronal cells

**Figure 2:**
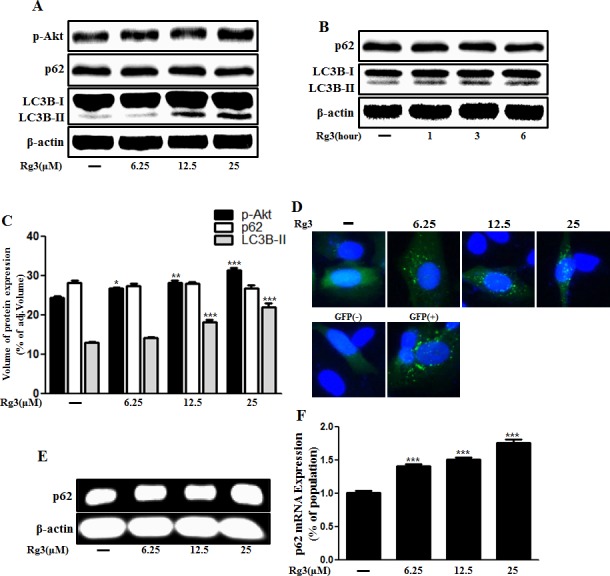

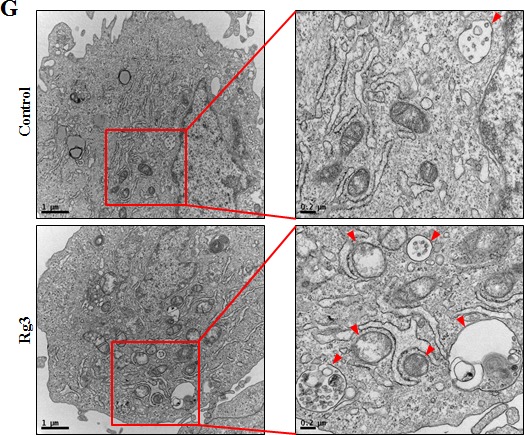
Rg3 increases the conversion to LC3B-II and autophagosomes in neuronal cells Primary neurons were treated with 6.25, 12.5, or 25 μM of Rg3 for 6 h (**A**) and 25 μM of Rg3 for 1, 3, 6 hour (**B**). Treated cells were assessed for LC3B production and p62 by Western blot analysis. (**C**) Bar graph indicating the averages of p-Akt, p62 and LC3B-II expression levels. (**D**) SK-N-SH cells were mixed with a titration (30MOI) of BacMam GFP-LC3B virus over 18 h and then treated with Rg3 at different concentrations for 6 h. Negative control reagent and positive control (CQ) were included at the same time. (**E**) RT-PCR for p62/SQSTM1 gene in primary neuron cells. (**F**) Real-time PCR for p62/SQSTM1 gene in primary neurons. (**G**) SK-N-SH cells were incubated with 25 μM of Rg3 for 6 h and analyzed by TEM. Arrowheads indicate autophagosomes. *** p < 0.001: significant differences between the control and each treatment group. GFP (+): Positive control; GFP (-): Negative control.

We hypothesized that autophagy flux could be the means by which Rg3 exerted its pro-survival effect against prion-induced neuronal damage. To test this hypothesis, we investigated whether Rg3 upregulated LC3B-II isoform of the LC3B, which is considered to be a marker protein for autophagy because LC3B-I isoform is converted to LC3B-II during the process of autophagosome formation [39]. We identified that the LC3B-II levels were higher in Rg3-treated group than in the control group (Figure 2A-2C). Premo™ Autophagy Sensor (LC3B-FP) BacMam 2.0 was employed to evaluate the activation of autophagy, as described previously [40]. As shown in Figure 2D, SK-N-SH cells treated with Rg3 had increased punctate fluorescence distribution pattern. Increase in p62/SQSTM1 mRNA expression, an indicator of autophagy flux induction, was detected in primary neurons (Figure 2E and 2F). Transmission electron microscopy (TEM) images of human SK-N-SH neuroblastoma cells revealed that Rg3 treatment induced the formation of double-membrane autophagosomes that contain entrapped cytoplasm or entire organelles (Figure 2G). These findings suggest that Rg3 could activate autophagy in human and mice neuronal cells.

### Rg3 inhibits PrP (106-126)-induced neuronal apoptosis via autophagy flux

**Figure 3:**
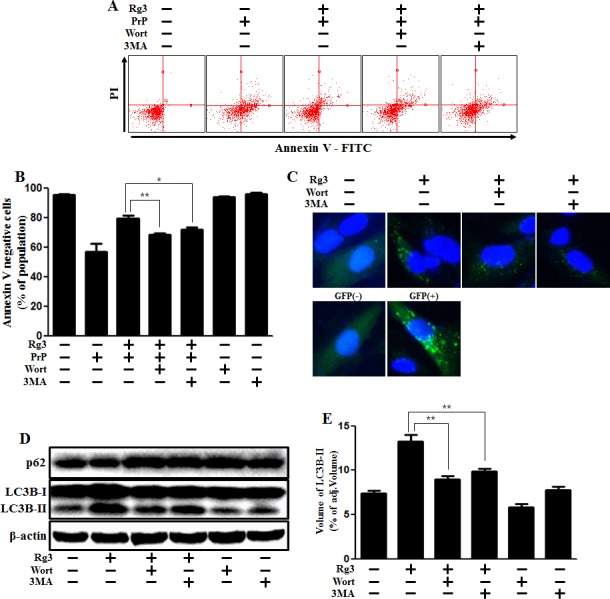

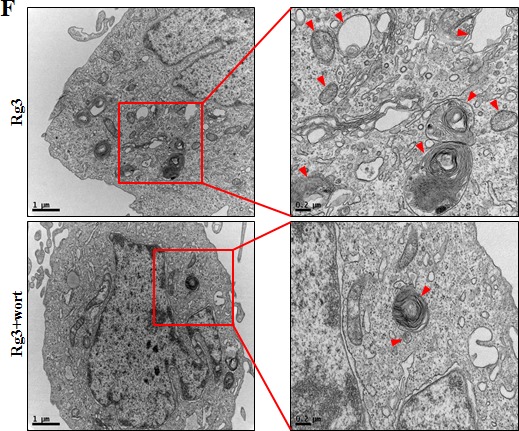
Rg3 protects neuronal cells via autophagic pathway (**A**) SK-N-SH neuroblastoma cells were pretreated with 25 μM of Rg3 in the presence of autophagy inhibitors (wortmannin or 3MA) for 6 h and then exposed to 100 μM of PrP (106-126) for 12 h. Cell viability was determined using Annexin V assay. (**B**) Bar graph indicating the averages of annexin V-negative cells. (**C**) SK-N-SH cells were mixed with a titration (30MOI) of BacMam GFP-LC3B virus for 18 h. They were then treated with Rg3 and autophagy inhibitors for 6h. Negative control reagent and positive control reagent (CQ) were included at the same time. (**D**) Western blot analysis for LC3B production and p62/SQSTM1 expression in SK-N-SH cells. Beta-actin was used as a loading control. (**E**) Bar graph indicating the averages of LC3B-II expression levels. (**F**) SK-N-SH cells were incubated with Rg3 at 25 μM and wortmannin for 6 h followed by TEM. Arrowheads indicate autophagosomes. *p < 0.05, **p < 0.01: significant differences between the control and each treatment group. PrP: Prion peptide (106-126); wort: wortmannin; 3MA: 3-Methyladenine; GFP (+): Positive control; GFP (-): Negative control.

Since the specific role of autophagy flux remains controversial, we aimed to determine whether autophagy flux has a neuroprotective function. We examined whether 3MA and wortmannin, the autophagy inhibitors, could reverse Rg3-mediated neuroprotective effects against PrP (106-126). The neuroprotective effect exerted by Rg3 decreased after treating the cells with the autophagy inhibitors (Figure 3A-3E). The previously observed increase of LC3B-II and green fluorescent puncta in SK-N-SH cells was diminished following exposure to autophagy inhibitors. In SK-N-SH cells, autophagy inhibitors caused a reduction in levels of LC-II expression by (Figure 3D and 3E). TEM images of neurons indicated that treatment of SK-N-SH cells with autophagy inhibitors resulted in a decrease of autophagosome (Figure 3F).

**Figure 4:**
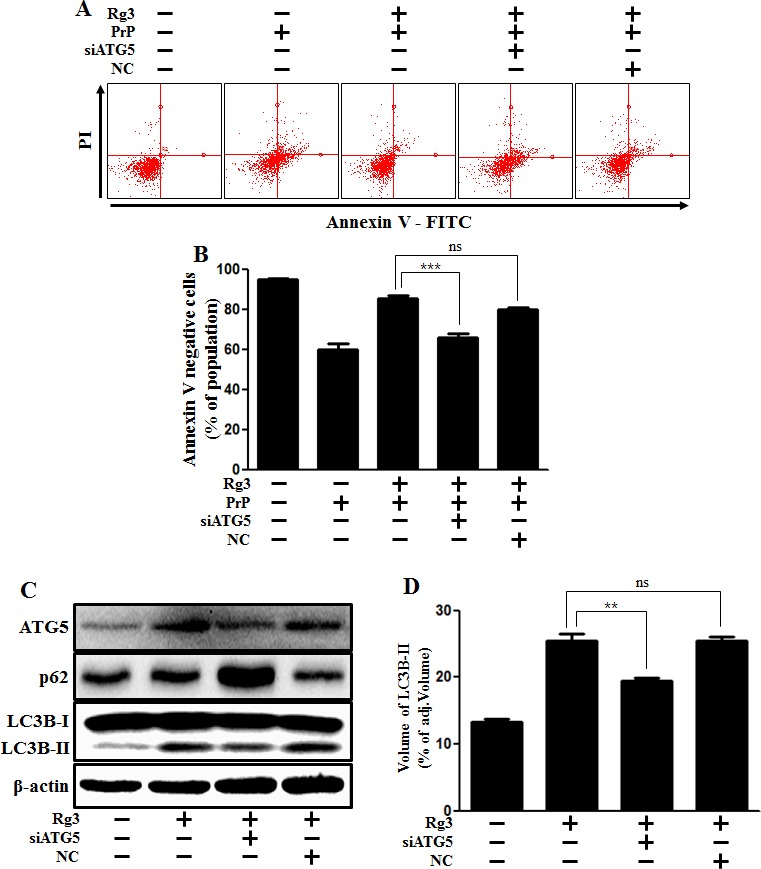
ATG5 knockdown decreases Rg3-mediated neuroprotective effect (**A**) ATG5 small interfering RNA (siATG5) or negative control siRNA (NC)-transfected SK-N-SH cells were incubated with PrP (106-126) for 12 h in the presence of Rg3. Cell viability was determined with Annexin V assay. (**B**) Bar graph indicating the averages of annexin V-negative cells. (**C**) Western blot analysis for ATG5 and LC3B production in SK-N-SH cells. Beta-actin was used as a loading control. (**D**) Bar graph indicating the averages of LC3B-II expression levels. **p < 0.01, *** p < 0.001: significant differences between the control and each treatment group. PrP: Prion peptide (106-126); NC: Negative control.

In addition, knocking down Atg5 diminished the neuroprotective effect mediated by Rg3 (Figure 4A and 4B) and decreased Rg3-induced autophagy in SK-N-SH cells (Figure 4C and 4D).

### Rg3 inhibits PrP (106-126)-induced mitochondrial damage via autophagy flux

**Figure 5:**
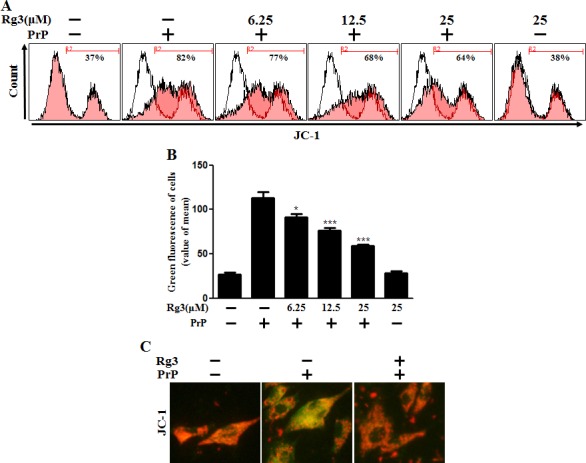
Rg3 inhibits PrP (106-126)-induced mitochondrial dysfunction in neuronal cells (**A**) Primary neurons were treated with 6.25, 12.5, or 25 μM of Rg3 for 6 h followed by incubation with PrP (106-126) for 12 h. Treated cells were used to measure JC-1 mono form (green) by flow-cytometry. R2 represents the population of JC-1 monomeric cells. (**B**) Bar graph indicating the averages of green fluorescent cells. (**C**) Representative images of J aggregate formation in SK-N-SH cells as described in A. *p < 0.05, *** p < 0.001: significant differences between the control and each treatment group.

**Figure 6:**
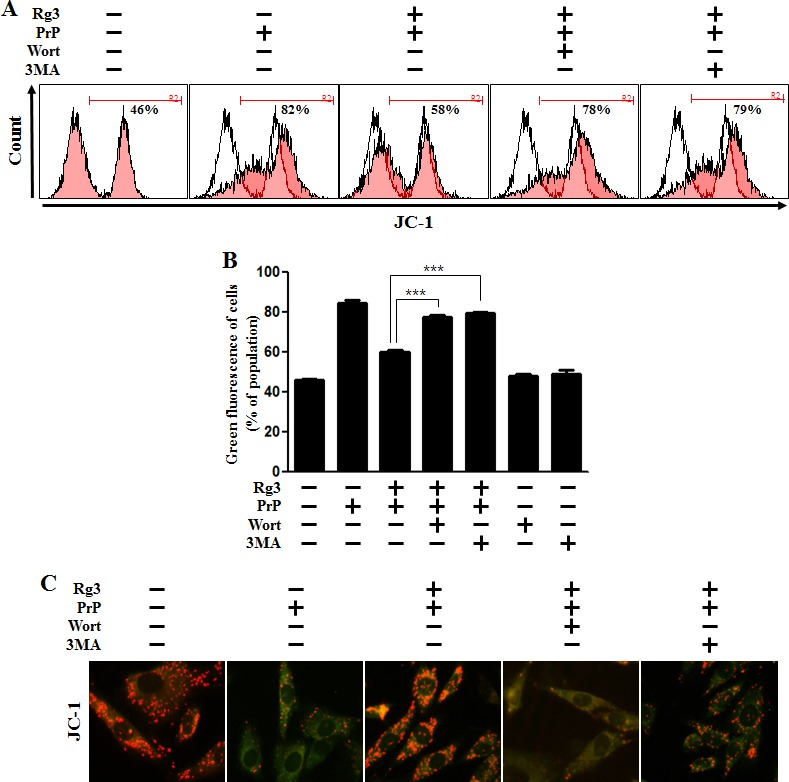
Rg3 recovers prion-induced mitochondrial dysfunction in neuronal cells via autophagy (**A**) Primary neurons were pretreated with 25 μM of Rg3 in the presence of autophagy inhibitors (wortmannin or 3MA) for 6 h and then exposed to 100 μM of PrP (106-126) for 12 h. Treated cells were used to measure the JC-1 mono form (green) by flow-cytometry. R2 represents the population of JC-1 monomeric cells. (**B**) Bar graph indicating the averages of green fluorescent cells. (**C**) Representative images of J aggregate formation in SK-N-SH cells as described in A. *** p < 0.001: significant differences between the control and each treatment group. PrP: Prion peptide (106-126); wort: wortmannin; 3MA: 3-Methyladenine.

We investigated whether Rg3 conferred neuroprotection mitochondrial damage induced by PrP (106-126). PrP (106-126) only treatment increased the level of JC-1 monomers meaning low MTP. However, treatment with Rg3 not only inhibited the PrP (106-126)-induced JC-1 monomer production but also increased the level of J-aggregate-form, an indicator of normal MTP (Figure 5A and 5B). Consistent with these results, in fluorescence microscopy images, PrP (106-126)-treated SK-N-SH cells were observed to have JC-1 monomer form (cells with green fluorescence), indicating lower MTP. However, both the negative control cells and Rg3-treated cells were observed to have JC-1 aggregate form (cells with red fluorescence), indicating high MTP values (Figure 5C). Our data demonstrated that Rg3 alleviated the mitochondrial dysfunction induced by prion protein peptides. Furthermore, based on the findings from flow cytometry in primary neuronal cells and fluorescence microscopy imaging of SK-N-SH cells (Figure 6A-6C), autophagy inhibitors blocked the protective effect of Rg3 against prion protein-induced mitochondrial damage. Overall, these researches indicate that Rg3 could prevent prion protein-induced mitochondrial damage via autophagy flux.

## DISCUSSION

This study showed that Rg3 could attenuate prion protein-induced neurotoxicity and mitochondrial dysfunction via autophagy flux. However, Rg3 alone was not sufficient to exert a biological effect in neurons. We believe this study will serve as a basis for further exploration of Rg3 and its effect.

It has already been suggested that, in some cases, Rg3 induced autophagy [41, 42]. Many researches have proposed that autophagy is shown to be a double-edged sword including beneficial and harmful effects [43, 44][44]. Autophagy regulates lysosomal degradation of intracellular long-lived or abnormal proteins and organelles to recycle subsequent under starvation and stresses [45, 46]. Usually, SQSTM1/p62 is degraded by autolysosomes when autophagy flux is activated. Some studies have suggested that SQSTM1/p62 has a protective role as a survival factor [47, 48]. SQSTM1/p62 is well-known autophagic substrate that has been widely used as a marker of autophagic degradation [49]. [49, 50]SQSTM1/p62 levels are typically inversely associated with lysosomal degradation via autophagy flux. Increase of p62-positive aggregates could be caused by loss of ATG genes those are required for process of autophagy flux [51, 52].

Although Atg5-dependent autophagy has been shown to be crucial for survival during the first few days of the starvation period [53], recent evidence has identified an alternative, Atg5-independent pathway of autophagy [54]. Knock-down of Atg5 is well used in many types of experiments since Atg5 is involved in autophagy, such as Atg5-Atg12 conjugation [12]. Therefore, we employed Atg5 siRNA as a tool to inhibit autophagy in neurons (Figure 4).

Reactive oxygen species (ROS), cytochrome c, apoptosis related factors released by damaged mitochondria those can cause damage to the entire cell [55]. Elimination of some dysfunctional mitochondria by autophagy flux should be able to preserve cellular homeostasis. Autophagic degradation of mitochondria occurs following both induction [56] and inhibition of apoptosis for cell survival [57]. Lemasters’ group demonstrated that autophagic induction by serum starvation can segregate depolarizing mitochondria by autophagic vacuoles [58, 59]. Here, we demonstrated that autophagy flux could undermine prion protein-induced mitochondria injury, suggesting that autophagy is closely associated with a mitochondria-related ion channel.

Akt is extremely important kinase in the management of cell survival. Akt signaling is activated in many kinds of malignant tumors and is related to the regualtion of cell development and survival [60]. Akt inhibits the activity of the TSC1/TSC2 (proteins harboring mutations in tuberous sclerosis) complex, a negative regulator of mammalian target of rapamaycin (mTOR), which regulates autophagy [12]. According to the experimental results, Rg3 treatment stimulated Akt signaling (Figure 2A and 2B), indicating that Rg3 enhanced neuronal cell survival. It also means that autophagy was inhibited if Akt was upstream of autophagy pathway. In this case, Akt was not upstream of autophagy and there is no relation between them (data not shown). Then, we considered that Akt might act as a survival factor in neurons instead of functioning as an upstream regulator of autophagy.

We demonstrated that Rg3 could induce autophagy flux, the main neuroprotective mechanism in prion disease as a therapeutic target. Because PrP (106-126) is not similar to PrPSc, we could only confirm PrPSc toxicity. The prion peptide 106-126 sequence is a practical model for *in vitro* study of prion-induced cell death, in addition to in vivo retinal neuron models treated with intravitreous injection of PrP fragments [61, 62]. Further studies are merited to determine the neuroprotective effect of Rg3 and autophagy flux in mouse models. Therefore, we shall further investigate the neuroprotective effect of Rg3 in a PrP (106-126)-induced mouse model and examine its potential therapeutic role as a drug in prion disease.

## MATERIALS AND METHODS

### Cell culture

The primary neurons and human neuroblastoma cell line SK-N-SH cells were prepared and method of cell culture has been described previously [41]. Primary neurons were isolated from brain of embryonic mice. SK-N-SH was obtained from the ATCC (Rockville, MD, USA).

### PrP (106-126) treatment

Synthetic PrP (106-126) peptides (sequence: Lys-Thr-Asn-Met-Lys-His-Met-Ala-Gly-Ala-Ala-Ala-Ala-Gly-Ala-Val-Val-Gly-Gly-Leu-Gly) were synthesized by Peptron (Seoul, Korea). These peptides were dissolved in sterile dimethyl sulfoxide at a stock concentration of 10 mM and stored at -20°C.

### Annexin V assay

Primary neuronal cells were treated Rg3 and PrP (106-126) indicated in Figure Legend. Apoptosis and cell death in detached cells was evaluated using annexin V assay kit as described previously [40].

### Terminal deoxynucleotidyl transferase dUTP nick end labeling (TUNEL) assay

TUNEL analysis was performed to measure the degree of cellular apoptosis as described previously [41].

### Trypan blue exclusion assay

Trypan blue dye exclusion (Sigma-Aldrich) was conducted to evaluate the number of viable cells using a hemocytometer. The result was expressed as a percentage of viable cells relative to vehicle-treated controls.

### Mitochondrial transmembrane potential assay

Neuron primary cells and SK-N-SH cells were incubated in media containing 10 μM JC-1 at 37°C for 15 min, washed with PBS, and transferred to a clear 96-well plate. The JC-1 aggregate fluorescence was measured at emission wavelength of 583 nm and excitation wavelength of 488 nm. JC-1 monomer fluorescence intensity was measured with an excitation of 488 nm and an emission wavelength of 525 nm using a Guava easyCyte HT System (Millipore). J-aggregates in intact mitochondria were evident as red fluorescence with emission at 583 nm, indicating high or normal MTP. Green fluorescence with emission at 525 nm indicates low MTP when JC-1 remains in the monomeric form in the cytoplasm. SK-N-SH cells were cultured onto cover slips in a 24-well plate, incubated in media containing 10 μM JC-1 at 37°C for 15 min, and washed with PBS. Finally, cells were mounted with DakoCytomation fluorescent medium (Dako, Carpinteria, CA, USA) and visualized under a fluorescence microscope.

### BacMam transduction

SK-N-SH cells were treated with GFP-tagged LC3B reagent for 18-24h and then treated Rg3 according to the manufacturer's instructions as described previously [41].

### RNA interference

Primary neuronal cells were transfected with ATG5 small interfering RNA (siRNA: oligoID HSS114104: Invitrogen, Carlsbad, CA, USA) and HIF-1α siRNA (oligoID HSS104775: Invitrogen) using transfection reagent according to the manufacturer's instructions as described previously [63].

### Western blot analysis

Western blot analysis was implemented using primary neuronal cells and SK-N-SH cells as described previously [40]. Primary antibodies used for immunoblotting were anti-LC3B (#4108, Cell Signaling Technology), anti-P62 (#5114, Cell Signaling Technology), anti-ATG5 (#2630, Cell Signaling Technology), and anti-β-actin (A5441, Sigma Aldrich).

### Quantitative real-time polymerase chain reaction (qRT-PCR)

Quantitative real-time polymerase chain reaction was implemented using primary neuronal cells as described previously [40]. The following primers were used: p62/SQSTM1 (forward: 5′CTCCCCAGACTACGACTTGTGT3′, reverse: 5′TCAACTTCAATGCCCAGAGG3′) and β-actin (as an internal control) (forward: 5′GCAAGCAGGAGTATGACGAG3′, reverse: 5′CAAATAAAGCCATGCCAATC3′).

### Transmission electron microscopy analysis

SK-N-SH cells were treated Rg3 indicated in Figure Legend. After fixation of samples in 2 % glutaraldehyde (EMS, USA) and 2 % paraformaldehyde (EMS, USA) in 0.05 sodium carcodylate buffer (pH7.2) (EMS, USA), specimens were post fixed in 1% osmium tetroxide (EMS, USA), dehydrated in graded ethanol and propylene oxide (EMS, USA). The cells were embedded in Epoxy resin (Embed 812, NMA; Nadic methyl anhydride, DDSA; Dodenyl Succinic Anhidride, DMP-30) (EMS, USA). Ultrathin sections were cut on an LKB-III ultratome (LEICA, Austria) and were stained with 0.5% uranyl acetate (EMS, USA) and lead citrate (EMS, USA). The images were taken on a Hitachi H7650 electron microscope (Hitachi, Japan) at an accelerating voltage of 100 kV.

### Statistical analysis

Unpaired t-tests or Welch's corrections were used to compare differences between two groups. One-way analysis of variance followed by Dunnett's test was used for multiple comparisons. All statistical analyses were performed with GraphPad Prism software (La Jolla, CA 92037 USA). Results were considered as significant at * p < 0.05, ** p < 0.01, or *** p < 0.001.
